# Soil As Levels and Bioaccumulation in *Suaeda salsa* and *Phragmites australis* Wetlands of the Yellow River Estuary, China

**DOI:** 10.1155/2015/301898

**Published:** 2015-01-21

**Authors:** Junjing Wang, Junhong Bai, Zhaoqin Gao, Qiongqiong Lu, Qingqing Zhao

**Affiliations:** State Key Laboratory of Water Environment Simulation, School of Environment, Beijing Normal University, Beijing 100875, China

## Abstract

Little information is available on As contamination dynamics in the soil-plant systems of wetlands. Total arsenic (As) in soil and plant samples from *Suaeda salsa* and *Phragmites australis* wetlands was measured in the Yellow River Estuary (YRE) in summer and autumn of 2007 to investigate the seasonal changes in As concentrations in different wetlands. The results showed that soil As levels greatly exceeded the global and regional background values. As levels in soil and the roots and stems of both types of plants were much higher in summer than in autumn, whereas leaf As showed higher level in autumn. Soil sulfur was the main factor influencing As levels in *Suaeda salsa* wetlands, whereas soil porosity was the most important factor for *Phragmites australis* wetlands. The contamination factor (CF) showed moderately to considerably polluted levels of As in both wetland soils. Plant roots and leaves of *Suaeda salsa* had higher As concentrations and biological concentration factors (BCFs) than stems, while the leaves and stems of *Phragmites australis* showed higher As levels and BCFs than roots. Compared to *Phragmites australis*, *Suaeda salsa* generally showed higher translocation factor (TF), while TF values for both plant species were higher in summer than in autumn.

## 1. Introduction

Arsenic (As) is ubiquitous in the natural environment [1] due to its natural and anthropogenic origins such as oil exploration, industrial emissions, the applications of insecticides and fertilizers, and sewage irrigation with As contaminated waste [2, 3]. Soil As pollution has posed a serious threat to the ecosystems due to its carcinogenic, mutagenic, and teratogenic effects [4, 5] and chronic toxicity [6], in particular its higher toxicity to the biota [7]. Moreover, soil As can do harm to human health through biological accumulation in the food chain. Therefore, soil As contamination in the ecosystems has been given increasing attention worldwide [8].

Wetland soils serve as source, sink, and transfer of chemical contaminants [9–11]. The retention time of these contaminants (e.g., As) in wetlands could be increased through wetland plants and water flow control, and then it effectively reduces the contaminant diffusion to the surrounding environment via various physical, chemical, and biological processes [12]. Most researchers have focused on As pollution characteristics in wetland soil/sediment [3, 5, 12, 13] and their influencing factors such as soil properties (e.g., SOM, pH value, and Eh) [14, 15] and anthropogenic activities (e.g., wetland reclamation, application of fertilizers, and sewage discharge) [13, 15]. Roychoudhury [16] investigated spatial and seasonal variations in depth profile of total As concentrations in salt marshes and found few variations in As concentrions along soil profiles in different seasons under natural conditions. However, Bai et al. [5] presented that soil As levles increased from spring to autumn in tidal wetlands due to the flow-sediment regulation of upstream Xiaolangdi reservior. Therefore, it is necessary to investigate and monitor the dynamic variations in As levels in these wetlands with strong hydrological fluctuations to lower As contamination risk. However, few studies have been carried out on As contamination dynamics in the soil-plant systems of wetland ecosystems, especially in estuarine wetlands.

The Yellow River Estuary is one of the larger estuaries in China, which is seriously affected by intense human activities such as the exploration of Shengli oilfield and the flow-sediment regulation of upstream Xiaolangdi reservior [5]. The primary objectives of this study were (1) to investigate the dynamic changes in As levels in soil and plant in estuarine wetlands with different plant communities (i.e.,* Suaeda salsa* and* Phragmites australis*) in the YRE; (2) to assess wetland plant's translocation and enrichment capacities and identify their influencing factors.

## 2. Materials and Methods

### 2.1. Study Area

The Yellow River Estuary (YRE), located on the south side of Bohai Sea, is one of the most active land-ocean interaction zones among the larger estuaries in the world, and it is also called the “Golden Triangle” due to its great exploitation potential and development [17]. It has a warm temperate monsoon climate with the annual average precipitation of 596.9 mm and the annual average air temperature of 12.9°C [18]. Most coastal wetlands have been suffering from serious degradation due to less freshwater inputs and intense anthropogenic activities. Since 2002, the flow-sediment regulation has been implemented from June to July of every year by the Yellow River Conservancy Commission to control the water and sediment discharge from the upstream Xiaoliangdi Reservoir [19]. The regulation regime has caused As and heavy metal pollution in the tidal freshwater wetlands and tidal salt marshes [5]. Moreover, the spillage and transportation of petroleum from Shengli oil field also brought serious soil contamination in this region since the oil exploitation in 1964 [20].

### 2.2. Sample Collection and Analysis

**Figure 1:**
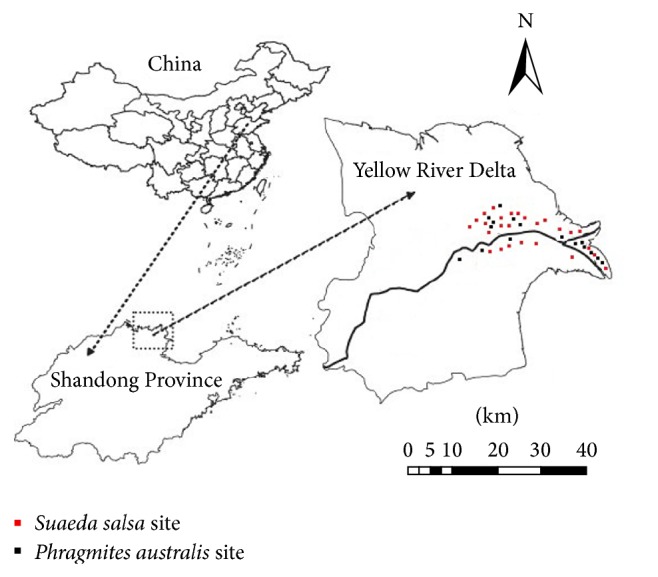
Location map of sampling sites in* Phragmites australis* and* Suaeda salsa* wetlands of the Yellow River Delta.

All sampling sites are located in the Yellow River Delta National Nature Reserve. The vegetation type and distribution pattern are dominantly controlled by water and salinity gradients in the YRE. The predominant vascular plants are* Phragmites australis* and* Suaeda salsa* [21]. Top 20 cm soils were sampled in both* Phragmites australis* and* Suaeda salsa.* In total, 16 soil samples from* Phragmites australis* wetlands and 25 soil samples from* Suaeda salsa* wetlands were collected in each of both seasons such as summer and autumn of 2007 in the YRE (Figure 1).

All soil samples were placed in polyethylene bags and transported to the laboratory and then air-dried at room temperature for three weeks. The air-dried soil samples were sieved through a 2 mm nylon sieve to remove the coarse debris and stones and then ground to fine powder and passed through a 0.149 mm nylon sieve. Another soil core (100 cm^3^) with three replicates for each sampling site was collected for the determination of soil moisture and bulk density. Meanwhile, plant samples with three replicates were also collected in 0.5 m × 0.5 m plots at each soil sampling site and plant root, stem, and leaf were separated from the whole plant. In total 48 plant samples (including root, stem, and leaf) of* Phragmites australis* and 75 plant samples of* Suaeda salsa* were taken. Plant samples were placed in paper bags after clean washing and then transported to the laboratory. All plant samples were oven-dried at 65°C for 48 h and ground into fine powder.

Soil and plant samples were, respectively, digested with an HClO_4_-HNO_3_-HF mixture and an HNO_3_-HClO_4 _mixture in Teflon tubes to analyze total concentrations of As in soil and plant. The solutions of the digested samples were determined using the inductively coupled plasma-atomic absorption spectrometry. In the meantime, soil phosphorus (P) and sulfur (S) concentrations in the digested samples were also determined using the same method [5]. Quality assurance and quality control were assessed using duplicates, method blanks, and standard reference materials (GBW07401 for soil and GBW 07602 for plant) from the Chinese Academy of Measurement Sciences with each batch of samples (1 blank and 1 standard for each 10 samples). The recovery of sample spiked with standards ranged from 95% to 99.88%.

Soil organic matter (SOM) was measured using dichromate oxidation method [22]. Soil pH was measured using a Hach pH meter (Hach Company, Loveland, CO, USA) (soil : water = 1 : 5). Salinity was determined in the supernatant of 1 : 5 soil-water mixtures using a salinity meter (VWR Scientific, West Chester, Pennsylvania, USA). The fresh soils were oven-dried at 105°C for 24 h and weighed for the determination of soil bulk density (BD) and moisture. Soil porosity was obtained from the difference between 1 and the ratio of BD to particle density (2.65 g/cm^3^).

### 2.3. Contamination Factor (CF)

As pollution in wetland soils was assessed using the contamination factor (CF) [23]. The CF is the ratio of the measured As concentration in the soil (*C*_measured_) to the background baseline value (*C*_background_). In this study, As background concentration (10.7 mg/kg) was obtained based on the environmental background concentrations of the loess materials of the Yellow River [24]. The formula was given:

(1) $$\begin{array}{c} \text{CF}=\frac{C_{\text{measured}}}{C_{\text{background}}}. \end{array}$$

According to Håkanson [23], CF values can be classified into four categories: (a) CF < 1, low contamination factor; (b) 1 ≤ CF < 3, moderate contamination factor; (c) 3 ≤ CF < 6, considerable contamination factor; (d) CF ≥ 6, very high contamination factor.

### 2.4. Biological Concentration Factor (BCF) and Translocation Factor (TF)

Biological concentration factor (BCF) and translocation factor (TF) are widely used to assess the ability of different plant tissues assimilating trace elements from soil and their translocation abilities from roots to aboveground plant tissues, respectively. The BCF and TF formulas are given as follows [25, 26]:

(2) $$\begin{array}{c} \text{BCF}=\frac{C_{\text{plant}\;\text{tisssue}}}{C_{\text{soil}}}, \end{array}$$

where the *C*_plant  tissue_ (mg/kg) is As concentration in plant tissue (i.e., stems, leaves, and roots) and *C*_soil_ (mg/kg) is As concentration in soil. Consider

(3) $$\begin{array}{c} \text{TF}=\frac{C_{\text{abovegound}}}{C_{\text{root}}}, \end{array}$$

where *C*_aboveground_ (mg/kg) and *C*_root_ (mg/kg) are As concentrations in the aboveground plant tissues and plant roots, respectively.

### 2.5. Soil As Storage

Soil As storage (kg As/ha) can be estimated by the following formula:

(4) $$\begin{array}{c} \text{SAsS}=0.1\times T\times \text{BD}\times \text{SAsC}, \end{array}$$

where SAsS is soil As storage; *T* is soil depth (cm); BD is bulk density (g/cm^3^); and SAsC is soil As concentration (mg/kg) in the given soil layer.

### 2.6. Statistical Analysis

Pearson correlation analysis was performed to identify the relationships among As concentrations, As storage, and selected soil properties. One-way ANOVA was implemented to test the differences in plant As, soil As, and selected soil properties between both sampling sites or between both two seasons. Differences were considered to be significant if *P* < 0.05. Statistical analysis was conducted using SPSS 16.0 for Windows (SPSS München, Germany) and Microsoft Excel 2012 software packages.

## 3. Results and Discussion

### 3.1. Soil Characterization in* Phragmites australis* and* Suaeda salsa* Wetlands

Selected properties of the top 20 cm soils in both* Phragmites australis* and* Suaeda salsa* wetlands in summer and autumn are summarized in Table 1. As shown in Table 1, soil pH values in* Suaeda salsa* wetlands were significantly higher in summer than those in autumn (*P* < 0.05), whereas soil pH in* Phragmites australis* wetlands did not show significant differences between both seasons (*P* > 0.05). Lower soil pH values in* Suaeda salsa* wetlands in autumn were probably attributed to the decreasing root activities and water content [27]. Soils in both wetlands had much higher porosity in summer compared to autumn (*P* < 0.05). Soil sulfur contents in* Phragmites australis* wetlands (528.87~554.20 mg/kg) were significantly lower than those in* Suaeda salsa* wetlands (609.31~687.11 mg/kg) (*P* < 0.05). Zeng et al. [28] presented that soil sulfur level increased with the increasing flooding frequencies, as the pioneer plant,* Suaeda salsa,* can suffer from more flooding frequencies than* Phragmites australis*. The average levels of soil moisture, BD, salinity, SOM, and total phosphorus (TP) did not show significant differences between* Phragmites australis* and* Suaeda salsa* wetlands and between both seasons.

### 3.2. Soil As Contamination Level

The mean As concentration ranged from 28.48 to 36.22 mg/kg in* Phragmites australis* and* Suaeda salsa* wetland soils with lower spatial variability (Table 1). The coefficients of variation of As levels in* Suaeda salsa* wetlands ranged from 6.21% to 12.75%, whereas they varied from 5.68% to 11.73% in* Phragmites australis* wetlands. All soil samples showed higher As levels exceeding the world guideline (10 mg/kg) [29], of which approximately 68.29% of soil samples had more than three times higher As levels than the worldwide guideline value. Meanwhile, As levels in all soils samples were also much higher than the background value of the loess materials of the Yellow River [24].* Suaeda salsa* wetland soils contained significantly higher As storage than* Phragmites australis* wetland soils in autumn (*P* < 0.05), whereas no significant differences in soil As storages (SAsS) were observed between* Phragmites australis* and* Suaeda salsa* wetlands in summer (*P* > 0.05). In* Phragmites australis* wetlands, SAsS exhibited a great decrease by approximately 40% in autumn compared to summer (*P* < 0.05; Table 1). Despite no significant differences in SAsS between summer and autumn in* Suaeda salsa* wetlands, a decreasing tendency was also observed from summer to autumn.

**Figure 2:**
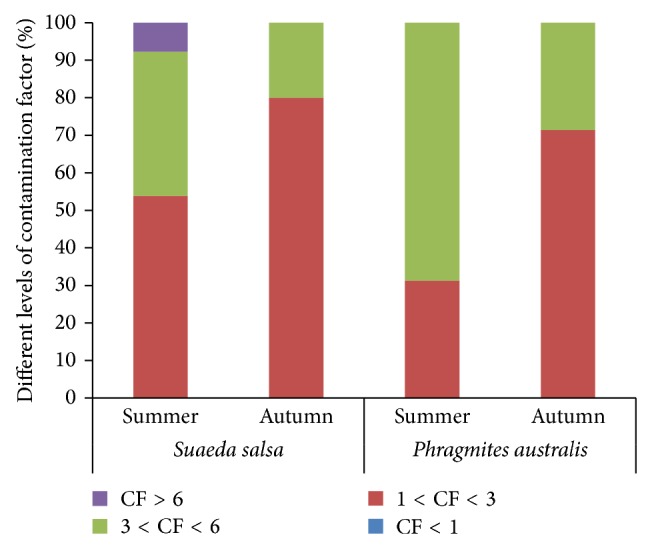
The percentage of different levels of contamination factors (CFs) of soils in* Phragmites australis* and* Suaeda salsa* wetlands in summer and autumn.

Contamination factor (CF) was defined as the ratio of As concentration in each sample to the background value [30]. As shown in Figure 2, all CFs of these soil samples exceeded 1, indicating that all sampling sites were suffering from As contamination. As contamination level is higher in summer than in autumn. More than 40% of soil samples from* Phragmites australis* wetlands and more than 60% of soil samples from* Suaeda salsa* wetlands showed considerable contamination levels with CFs values exceeding 3 in summer, whereas the CFs values of more than 70% of soil samples were less than 3 in autumn. Table 1 also showed that the mean soil As levels were significantly higher in* Phragmites australis* and* Suaeda salsa* wetlands in summer compared to autumn. Meanwhile, we observed that approximately 7% of soil samples from* Suaeda salsa* wetlands exhibited very high contamination level, implying that some* Suaeda salsa* wetlands were facing very high As contamination risks. Further studies were still needed to investigate the pollution sources at these sites. Therefore, the coastal wetland soils in the YRE were suffering from high As contamination risk. This might be associated with As accumulation and retention in this region due to natural and anthropogenic activities, such as oil field exploration in the YRE and the applications of pesticides and fertilizers in the upstream agricultural areas [3, 31, 32]. Bai et al. [5] presented that the flow-sediment regulation regime could bring more As to wetland soils in the YRE. Additionally, Rotkin-Ellman et al. [33] reported that flooding might be one important factor influencing soil As contamination in wetlands.

### 3.3. Relationships between Soil As and Selected Other Soil Properties

Bai et al. [34] and Farooq et al. [35] presented that SOM could play an important role in affecting soil As contents because SOM could adsorb As and decline greatly its mobility [32]. However, no significant correlations were observed between As and SOM in this study (Table 2). Moreover, As concentration and storage in both wetlands showed a weak negative correlation with SOM, which was associated with higher soil pH values, as As would exhibit higher mobility at higher pH range [14]. This was because the anaerobic carbon decomposition is likely to produce slowly in the flooded areas [36] and thus affected the mobilization of As. Total S contents were significantly correlated with As concentration and storage in* Suaeda salsa* wetlands (*P* < 0.05), whereas no significant correlations were observed between them in* Phragmites australis* wetlands (*P* > 0.05). The possible explanation is that sulfur might improve plant accumulation capacity of* Suaeda salsa* to As through improving tolerance caused by a positive effect on thiol metabolism and antioxidant status of plants [37], which could lead to a decrease in soil As concentrations. Higher As concentrations were also observed in plant tissues of* Suaeda salsa* compared to* Phragmites australis* (Table 3). However, in* Phragmites australis* wetlands, As concentration and storage were significantly and positively correlated with soil porosity (*P* < 0.05), which was highly associated with the fact that As (III) can be oxidized to As (V) with lower toxicity and bioavailability [38], thus improving As accumulation in soil. Additionally, soil pH values and salinity did not show significant effects on soil As due to similar pH values and salinity in these sampling sites.

### 3.4. Levels, Accumulation, and Translocation of Plant As

As concentrations in various plant tissues (root, stem, and leaf) of* Phragmites australis* and* Suaeda salsa* in summer and autumn are listed in Table 3. As concentrations in plants varied with plant species and plant tissues [2]. The leaves of* Phragmites australis* had the highest As concentration, followed by stems, whereas roots showed the lowest As levels in both seasons. Tsutsumi [39] reported that the absorbed As by root could be transported to plant aboveground parts, causing elevated concentration of leaf As. As for* Suaeda salsa*, leaves and roots showed higher As concentrations than stems. This is consistent with the results by Madejón et al. [40] and Smith et al. [31], who presented that As accumulation mainly occurred in roots. Generally, almost all plant tissues of* Suaeda salsa* had higher As concentrations compared to* Phragmites australis* in both seasons except for lower stem As levels in autumn. As concentrations in roots and stems of both* Phragmites australis* and* Suaeda salsa* generally decreased from summer to autumn, which might be caused by As translocation from root to stem and leaf with higher As concentrations in autumn than in summer. Moreover, higher soil As concentrations in summer also contributed to improving plant As levels, as As absorption and accumulation by plants were significantly and positively correlated with soil As [25, 41].

**Figure 3:**
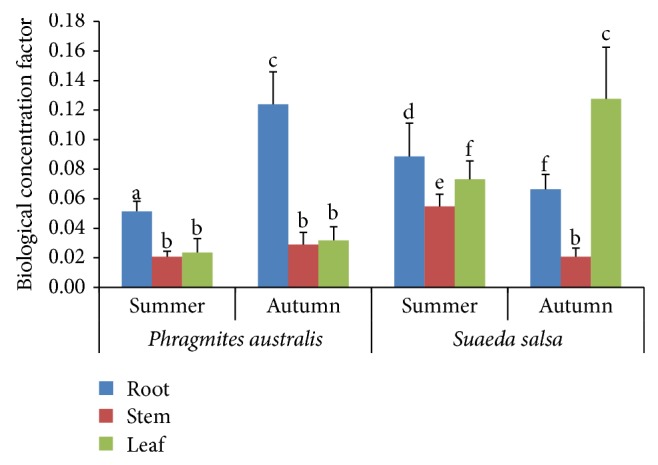
Biological concentration factors (BCFs) of different plant tissues of both* Suaeda salsa* and* Phragmites australis* in summer and autumn in the YRE. Different letters (abcdef) represent significant differences in BCFs of different tissues (*P* < 0.05).

BCF and TF values could be used to evaluate the potential capacities of plant species for phytoextraction and phytostabilization [2]. As for* Suaeda salsa*, the roots and stems showed higher BCFs in summer than in autumn, whereas lower BCFs were observed for the leaves (*P* < 0.05). In contrast,* Phragmites australis* roots showed significantly higher BCFs in autumn than in summer (*P* < 0.05); however, the BCFs of roots and stems of* Phragmites australis* exhibited similar lower BCFs in both seasons compared to leaves. Compared to* Phragmites australis*,* Suaeda salsa* had higher BCFs for all tissues in summer. This indicates that As accumulation in different plant tissues is associated with plant types and seasons (Figure 3).

**Figure 4:**
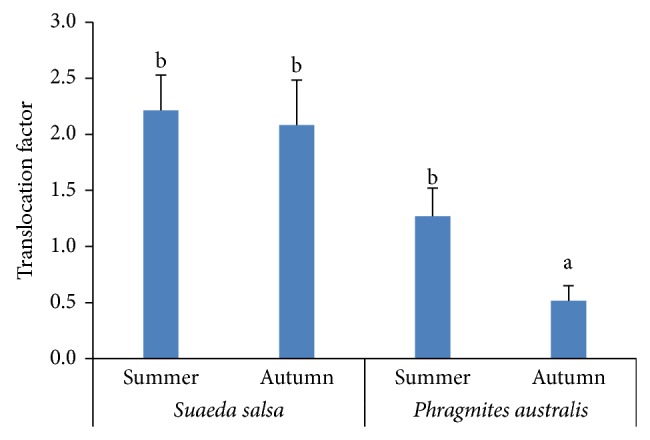
Translocation factors (TFs) of* Suaeda salsa* and* Phragmites australis* in summer and autumn. Different letters (ab) represent significant differences in TFs of* Phragmites australis* and* Suaeda salsa* in both seasons.

TFs of As for* Phragmites australis* and* Suaeda salsa* in both seasons are illustrated in Figure 4. TFs of both plant species were higher in summer compared to autumn. Generally,* Suaeda salsa* had higher TFs (>1) than* Phragmites australis* (<1) in both seasons. Therefore,* Suaeda salsa* had higher As-tolerance capability compared to* Phragmites australis* in this region, as* Suaeda salsa* could decrease As stress through translocating more As from roots to aboveground parts [32].

## 4. Conclusions

Soil As and plant As levels exhibit different distribution patterns. Generally, soil As, root As, and stem As are higher in summer than in autumn, while leaf As shows higher level in autumn for both* Phragmites australis* and* Suaeda salsa*. Soil S and porosity can influence soil As concentration and storage in both* Suaeda salsa* and* Phragmites australis* wetlands. All soil samples have a potential low or moderate risk of As contamination in the coastal wetlands of the Yellow River Estuary, so much more attention should be paid to controlling and monitoring As contamination level in this region, especially in summer.* Suaeda salsa* can be served as a native species to control the low or moderate levels of As contamination as* Suaeda salsa* exhibits higher bioaccumulation and translocation capability than* Phragmites australis.* Further studies on As forms and behaviors and the phytoremediation processes such as phytostabilization, phyroestraction, and phytotransformation of wetland plants (i.e.,* Suaeda salsa* and* Phragmites australis)* are still needed to remediate As contamination. Moreover, it is more helpful and meaningful to understand the tolerant threshold values of* Phragmites australis* and* Suaeda salsa* to As stress to maintain wetland ecosystem health.

## Figures and Tables

**Table 1:** Soil properties in both *Suaeda salsa* and *Phragmites australis* wetlands in summer and autumn.

	*Suaeda salsa* wetlands		*Phragmites australis* wetlands	
	Summer	Autumn	Summer	Autumn
Moisture (%)	25 ± 1.26^Aa^	25 ± 0.34^Aa^	27.49 ± 0.97^Aa^	25.43 ± 1.13^Aa^
BD (g/cm^3^)	1.83 ± 0.01^Aa^	1.84 ± 0.04^Aa^	1.82 ± 0.03^Aa^	1.81 ± 0.03^Aa^
Porosity	30.94 ± 0.22^Aa^	26.40 ± 0.34^Bb^	31.32 ± 0.68^Aa^	25.43 ± 0.27^Bb^
pH	8.42 ± 0.07^Aa^	7.91 ± 0.07^Bb^	8.36 ± 0.07^Aa^	8.29 ± 0.12^Ba^
Salinity (‰)	1.35 ± 0.24^Aa^	1.46 ± 0.27^Aa^	1.35 ± 0.47^Aa^	0.78 ± 0.43^Bb^
SOM (%)	6.06 ± 0.35^Aa^	7.17 ± 1.29^Aa^	5.60 ± 0.49^Aa^	6.89 ± 0.99^Aa^
As (mg/kg)	36.22 ± 2.25^Aa^	28.48 ± 3.63^Ab^	37.49 ± 2.13^Aa^	23.27 ± 2.73^Bb^
As storage (kg/ha)	133.02 ± 8.41^Aa^	104.70 ± 12.79^Aa^	137.18 ± 10.09^Aa^	83.80 ± 8.81^Bb^
TS (mg/kg)	609.31 ± 23.19^Aa^	687.11 ± 46.40^Aa^	554.20 ± 38.89^Ba^	528.87 ± 11.46^Ba^
TP (mg/kg)	674.48 ± 15.19^Aa^	627.97 ± 15.91^Ab^	657.31 ± 23.74^Aa^	612.18 ± 17.97^Aa^

BD: bulk density; SOM: soil organic matter; TP: total phosphorus; TS: total sulfur.

Different letters (AB) indicate significant differences in soil properties between *Phragmites australis *and *Suaeda salsa *in the same season.

Different numbers (ab) indicate significant differences in soil properties between summer and autumn in the same wetlands. SE means the standard error.

**Table 2:** Correlation coefficients among As, As storage, and selected soil properties.

		Moisture	BD	Porosity	pH	Salinity	SOM	TP	TS
*Suaeda salsa* wetlands	As	−0.269	0.028	0.331	0.264	−0.077	−0.243	−0.110	−0.537^**^
	SAsS	−0.287	0.094	0.325	0.248	−0.073	−0.257	−0.128	−0.549^**^
*Phragmitesaustralis *	As	−0.088	0.116	0.769^**^	0.344	0.110	−0.255	0.478	−0.124
wetlands	SAsS	−0.197	0.287	0.741^**^	0.326	0.122	−0.325	0.474	−0.198

BD: bulk density; SOM: soil organic matter; TP: total phosphorus; TS: total sulfur.

^**^Significant correlation at the 0.01 level (2-tailed).

**Table 3:** Seasonal changes in As concentrations (mean ± SE, mg/kg) in different plant tissues of *Suaeda salsa* and *Phragmites australis*.

	*Suaeda salsa *		*Phragmites australis *	
	Summer	Autumn	Summer	Autumn
Root	2.62 ± 0.46^a^	1.81 ± 0.26^b^	0.79 ± 0.15^c^	0.56 ± 0.12^c^
Stem	1.81 ± 0.28^b^	0.56 ± 0.14^c^	1.20 ± 0.31^e^	0.67 ± 0.16^c^
Leaf	2.27 ± 0.29^a^	3.16 ± 0.65^d^	1.86 ± 0.22^b^	2.82 ± 0.53^a^

Different letters (abcde) indicate significant differences in As concentrations between *Phragmites australis *and *Suaeda salsa*.

SE means the standard error.
